# Phase 1 study of intraventricular ^131^I-omburtamab targeting B7H3 (CD276)-expressing CNS malignancies

**DOI:** 10.1186/s13045-022-01383-4

**Published:** 2022-11-12

**Authors:** Kim Kramer, Neeta Pandit-Taskar, Brian H. Kushner, Pat Zanzonico, John L. Humm, Ursula Tomlinson, Maria Donzelli, Suzanne L. Wolden, Sophia Haque, Ira Dunkel, Mark M. Souweidane, Jeffrey P. Greenfield, Satish Tickoo, Jason S. Lewis, Serge K. Lyashchenko, Jorge A. Carrasquillo, Bae Chu, Christopher Horan, Steven M. Larson, Nai-Kong V. Cheung, Shakeel Modak

**Affiliations:** 1 Departments of Pediatrics, Memorial Sloan Kettering Cancer Center, 1275 York Avenue, Box 429, New York, NY 10065 USA; 2 Department of Radiology, Memorial Sloan Kettering Cancer Center, New York, NY 10065 USA; 3 Departments of Medical Physics, Memorial Sloan Kettering Cancer Center, New York, NY 10065 USA; 4 Departments of Radiation Oncology, Memorial Sloan Kettering Cancer Center, New York, NY 10065 USA; 5 Department of Neurosurgery, Memorial Sloan Kettering Cancer Center, New York, NY 10065 USA; 6 Departments of Pathology, Memorial Sloan Kettering Cancer Center, New York, NY 10065 USA

**Keywords:** CNS tumors, CNS metastases, Radioimmunotherapy, B7H3, Omburtamab

## Abstract

**Background:**

The prognosis for metastatic and recurrent tumors of the central nervous system (CNS) remains dismal, and the need for newer therapeutic targets and modalities is critical. The cell surface glycoprotein B7H3 is expressed on a range of solid tumors with a restricted expression on normal tissues. We hypothesized that compartmental radioimmunotherapy (cRIT) with the anti-B7H3 murine monoclonal antibody omburtamab injected intraventricularly could safely target CNS malignancies.

**Patients and methods:**

We conducted a phase I trial of intraventricular ^131^I-omburtamab using a standard 3 + 3 design. Eligibility criteria included adequate cerebrospinal fluid (CSF) flow, no major organ toxicity, and for patients > dose level 6, availability of autologous stem cells. Patients initially received 74 MBq radioiodinated omburtamab to evaluate dosimetry and biodistribution followed by therapeutic ^131^I-omburtamab dose-escalated from 370 to 2960 MBq. Patients were monitored clinically and biochemically for toxicity graded using CTCAEv 3.0. Dosimetry was evaluated using serial CSF and blood sampling, and serial PET or gamma-camera scans. Patients could receive a second cycle in the absence of grade 3/4 non-hematologic toxicity or progressive disease.

**Results:**

Thirty-eight patients received 100 radioiodinated omburtamab injections. Diagnoses included metastatic neuroblastoma (*n* = 16) and other B7H3-expressing solid tumors (*n* = 22). Thirty-five patients received at least 1 cycle of treatment with both dosimetry and therapy doses. Acute toxicities included < grade 4 self-limited headache, vomiting or fever, and biochemical abnormalities. Grade 3/4 thrombocytopenia was the most common hematologic toxicity. Recommended phase 2 dose was 1850 MBq/injection. The median radiation dose to the CSF and blood by sampling was 1.01 and 0.04 mGy/MBq, respectively, showing a consistently high therapeutic advantage for CSF. Major organ exposure was well below maximum tolerated levels. In patients developing antidrug antibodies, blood clearance, and therefore therapeutic index, was significantly increased. In patients receiving cRIT for neuroblastoma, survival was markedly increased (median PFS 7.5 years) compared to historical data.

**Conclusions:**

cRIT with ^131^I-omburtamab is safe, has favorable dosimetry and may have a therapeutic benefit as adjuvant therapy for B7-H3-expressing leptomeningeal metastases.

*Trial registration*: clinicaltrials.gov NCT00089245, August 5, 2004.

**Supplementary Information:**

The online version contains supplementary material available at 10.1186/s13045-022-01383-4.

## Background

Metastatic cancers prefer sanctuary sites such as the central nervous system (CNS), and the prognosis remains dire despite aggressive therapies [1, 2]. Compartmental radioimmunotherapy (cRIT) administering radiolabeled tumor-specific monoclonal antibodies (mAbs) could significantly improve the therapeutic index against low tumor burden in the leptomeninges while minimizing systemic radiation exposure [1–8]. We have previously reported on the feasibility of cRIT using ^131^I-labeled mAbs for CNS metastases in non-human primates [9] and in patients [10]. Unlike the relatively large blood volume (80–100 ml/kg; 5 L in adults), CSF volume is only 130–150 ml, allowing high drug concentrations even with small mAb doses [11]. Importantly, CSF flow is unidirectional with no recirculation, substantially reducing bystander toxicity to brain, spinal cord and spinal bone marrow (BM) [12]. Ironically, while unable to penetrate the CSF because of the blood–brain barrier, neutralizing human antibodies to mouse mAb accelerates blood clearance, reducing systemic exposure to vital organs [9].

Omburtamab (a murine IgG1 mAb previously named 8H9) has a high affinity for B7H3 (CD276), a tumor-associated surface immunomodulatory glycoprotein [13]. When radiolabeled with ^124^I, it becomes a theranostic agent, emitting positrons (e^+^) which permit high-resolution PET for accurate tumor imaging and precise determination of radiation dosimetry, while delivering β particle therapy either through the CSF, or through convection-enhanced delivery into the pons [14]. Unlike ^124^I-omburtamab, ^131^I-omburtamab (γ emitting) can only provide gamma or single-photon emission computed tomography (SPECT) images, although its β radiation is equally effective [15]. Both ^124^I-omburtamab and ^131^I-omburtamab are currently being investigated in cRIT for intraperitoneal metastases (clinicaltrials.gov NCT01099644) [16] and for pontine tumors (clinicaltrials.gov NCT01502917) [17]. To test the safety of ^131^I-omburtamab administered into the CSF compartment, a phase I dose escalation study was completed in patients with proven or suspected leptomeningeal metastases. Pharmacokinetics (PK) of radiolabeled 8H9 in serial CSF and blood samples was determined. The impact of human anti-mouse antibody (HAMA) on PK was also measured.

## Methods

### Study design and participants

An investigator-initiated, single-arm, single-center, phase I trial with a standard 3 + 3 dose escalation and expansion cohort design (NCT00089245) [18], the primary objective of which was to study the toxicity and to establish the recommended phase II dose for ^131^I-omburtamab, was performed. Secondary objectives included determining the biodistribution and dosimetry of intraventricular ^131^I-omburtamab. Study patients or legal guardians provided written informed consent.

Patients of any age with a high risk or recurrent primary or metastatic CNS malignancy known to express B7H3 (i.e., neuroblastoma (NB), retinoblastoma, medulloblastoma, rhabdomyosarcoma) [19] were offered participation. For tumors known to express B7H3, immunohistochemical assessment was not mandated; for other tumors, B7H3 expression on the cell surface was analyzed on frozen tumors using previously described methods [19]. For the latter, > 1 + results on immunohistochemistry were acceptable for enrollment. Additional eligibility criteria included stable neurologic status, no obstructive or symptomatic communicating hydrocephalus, absolute neutrophil count > 1000/µL, platelet count > 50,000/µL, serum bilirubin < 3.0 mg/dL, and serum creatinine < 2 mg/dL. Focal or craniospinal irradiation (CSI) or chemotherapy > 3 weeks before enrollment and ongoing corticosteroid therapy were allowed. An indwelling intraventricular access device (e.g., Ommaya catheter) was placed, and position, patency and cerebrospinal fluid (CSF) flow were evaluated by intraventricular ^111^In-diethylene triamine pentaacetic acid scan. Measurable or evaluable disease at study entry was not required. For patients who enrolled at > dose level 6, banked autologous hematopoietic stem cells of any quantity available for reinfusion if necessary were required.

### Treatment and toxicity assessment

After thyroid blockade with saturated potassium iodide oral solution and liothyronine for > 5 days patients received a dose of 74 MBq (2 mCi) ^124^I-omburtamab or ^131^I-omburtamab for dosimetry and biodistribution studies. This was followed about 7 days later by the therapeutic dose of ^131^I-omburtamab escalated in cohorts of 3 patients each. Doses were adjusted downward by 50% for patients ≤ 12 months old and by 33% for patients of 13 to 36 months old [20]. Premedications before each dose of omburtamab included dexamethasone, acetaminophen and diphenhydramine, administered routinely in anticipation of a possibly allergic reaction to the murine mAb (Additional file 3: Table S1). A specific activity or radioiodinated omburtamab was ~ 185 MBq/mg for the 2–50 mCi dose range and ~ 1850 MBq/mg for doses ≥ 50 mCi. Typically, a volume of 2–3 ml of drug was administered per injection, with close to an equal volume of CSF withdrawn. Outpatient and in-patient settings were both permitted for cRIT and MSK radiation safety precautions were strictly implemented including the use of distance and personal protective equipment, patient and caregiver education and dose rate monitoring using portable radiation detectors. The dose to family caregivers was maintained at < 500 millirem in accordance with applicable regulations. Thyroid blockers were continued for 2 weeks after the last mAb injection. HAMA in blood was tested using an enzyme-linked immunosorbent assay previously described before and generally 5 weeks after injection [21]. Patients were evaluated clinically on the day of each injection, days 1 and 2 post-injection, ~ 2 weeks and ~ 3 weeks post-injection. CBC and complete blood chemistry were tested on days of injection, during weeks 4 or 5 or earlier if clinically indicated. CSF was tested prior to each injection and during week 5 for protein, cell count and cytology.

### Toxicity assessment

Toxicity was assessed by clinical and biochemical evaluations and graded by the Common Terminology Criteria for Adverse Events version 3.0 over a 5-week period. Dose-limiting toxicity (DLT) observation period was 5 weeks after the first dose of radioiodinated omburtamab and was defined as non-hematologic grade 3 or 4 toxicity attributable to cRIT with the following exceptions: < grade 4, headache, fever, vomiting and hyperglycemia. Myelosuppression, while noted, was not considered DLT. If DLT was encountered in ≥ 1/3 patients at a given dose level, 3 more patients were accrued at that dose level. At dose levels 7 and 8, the cohort was increased to 6 patients enrolled at each dose level to better assess safety. In order to assess the cumulative toxicity of ^131^I-omburtamab, if patients did not experience DLT or unexpected grade 3 or 4 toxicity and did not have progressive disease, they could receive two further doses of radioiodinated omburtamab ≥ 4 weeks after the first dose: one at 74 MBq for dosimetry and the other at the cohort-specific therapeutic dose.

### Dosimetry evaluation

**Fig. 1:**
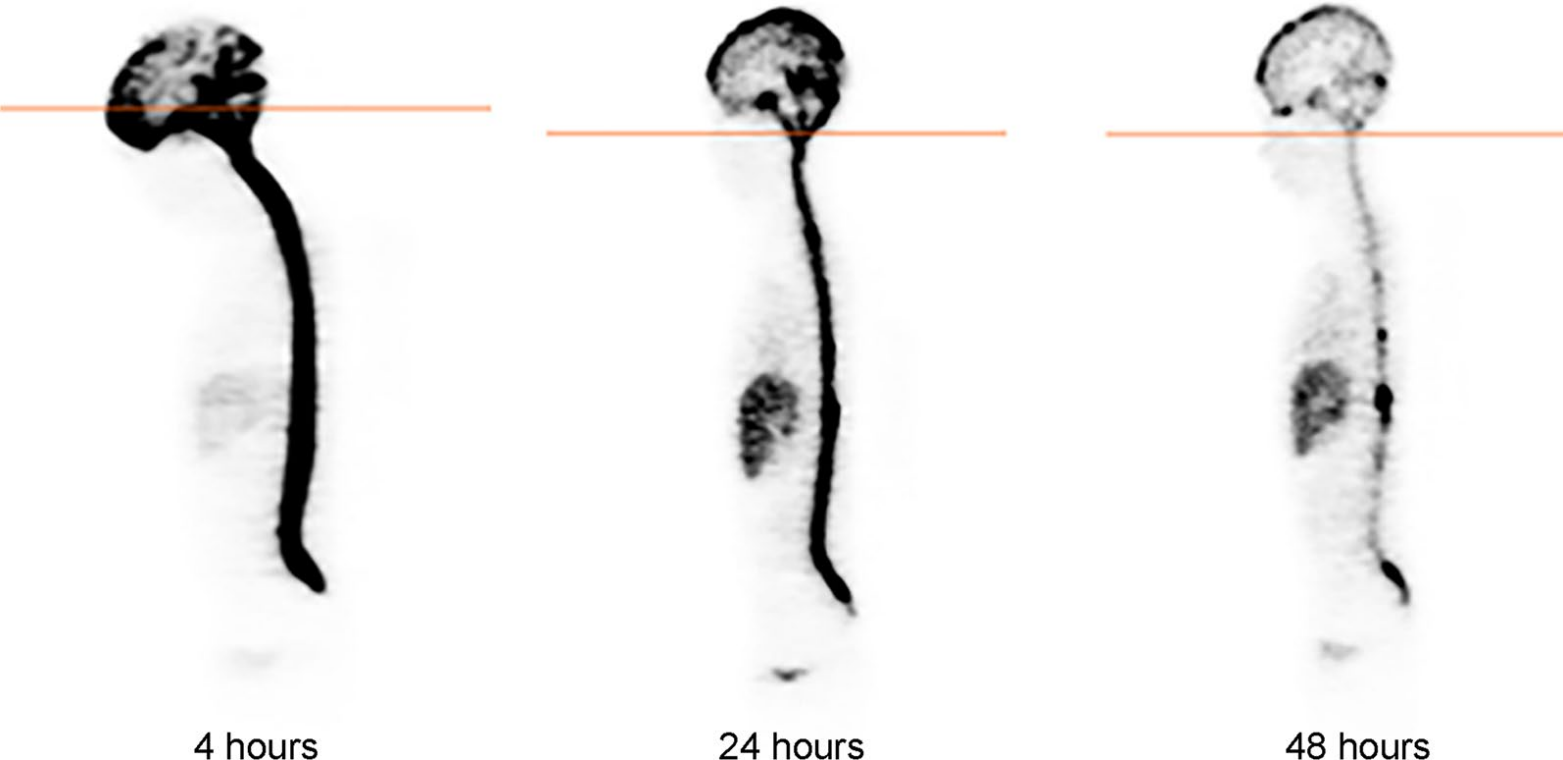
124-I-omburtamab whole-body positron emission tomography (PET) scans obtained approximately 4 h, 24 h and 48 h following a single injection of 74 MBq intraventricular 124-I-omburtamab, demonstrating activity throughout the ventricles, the sac and over the convexities

Dosimetry was measured by both CSF and blood sampling and by serial PET or SPECT scans. After administration of the initial 74 MBq dose CSF and blood were sampled at ~ 15, 30, 60, and 120 min and at ~ 2, 18, and 42–48 h after injection. Three PET or Gamma camera-SPECT scans, respectively, at approximately 4, 24 and 48 h [22] (Fig. 1). Measured CSF and blood aliquots were counted to estimate the time-dependent activity concentrations. The respective time–activity data were fit to exponential functions and integrated to yield the decayed area under the curves, representing cumulative CSF and blood activity [10]. Radiation dosimetry was done using previously described methods including delineating regions of interest (ROI) around various organs and calculating ^124^I or ^131^I time–activity area under the curve (AUC) for each ROI [7, 22]. Radiation absorbed doses to the ventricles of the CNS and the thecal sac were calculated using the *OLINDA* radionuclide dosimetry program, version 1.0 [22]. Radiation dosimetry calculations for the CSF, ventricles, spinal cord and blood were based on the assumption of complete local absorption of the beta radiation. For patients undergoing PET scans after IO 74 MBq ^124^I-omburtamab, projected absorbed doses to other organs for ^131^I-omburtamab were also estimated.

### Response assessment

For patients with NB, MIBG, CTCAP and bone marrow examinations were also performed approximately 1 month before and after cRIT. Responses were scored using International Neuroblastoma Response Criteria [23]. For other diagnoses, objective radiographic changes in the size of measurable disease or leptomeningeal enhancement from initial pre-^131^I-omburtamab treatment were noted. Progressive disease was defined as an increase in the size of a measurable lesion or the development of a new lesion. The Response Assessment in Neuro-Oncology Brain Metastases initiative [24] had not been implemented at study commencement and was therefore not utilized for assessing response.

### Statistical analysis

Radiation doses to the CSF as determined by serial PET/gamma camera scans were correlated with those determined by CSF sampling. The impact of human anti-mouse antibody (HAMA) and dose was assessed by Students t test comparisons. Progression-free survival (PFS) and overall survival (OS) were estimated using Kaplan–Meier statistics from the day of ^131^I-omburtamab administration and censored on February 1, 2020.

## Results

### Demographics and dose escalation

Thirty-eight (13 female; 25 male) patients were enrolled and received at least one dose of radioiodinated omburtamab. Diagnoses included neuroblastoma (*n* = 17), medulloblastoma (*n* = 6), ependymoma (*n* = 3), melanoma and rhabdomyosarcoma (*n* = 3 each), choroid plexus carcinoma (*n* = 2), and atypical rhabdoid teratoma, chordoma, pineoblastoma and retinoblastoma (*n* = 1 each). All patients had received at least one prior regimen of chemotherapy and radiotherapy before receiving cRIT. All patients with neuroblastoma except for 1 had received craniospinal irradiation prior to cRIT; the remaining patient had received cranial radiotherapy. Two patients with neuroblastoma were enrolled, received a dosimetry dose only and were subsequently removed, one each because of non-compliance and progressive disease, respectively. Of the remaining 36 patients, 19 and 17 received 1 and 2 cycles, respectively (Additional file 1: Fig. S1; Table 1). Most patients received cRIT in the outpatient setting. One patient received an additional imaging dose of 74 MBq due to concerns about catheter malfunction; CSF flow study was performed again before proceeding to a full therapeutic cycle. The median age at first cRIT injection was 6.6 (range 1.2–53.5 years). The starting therapeutic dose was 370 MBq (10 mCi), which was chosen based on our previous experience where 370 MBq was the maximal tolerated dose when IO radioiodinated anti-GD2 mAb 3F8 was used [10]. Diagnoses, dose at trial entry, number of injections and number of patients treated at each dose level are summarized in Table 1. 37% (11/30 tested) had positive HAMA titers prior to cRIT and 39% (14/36) had previously received murine mAbs. Two patients (1 each at dose level 1110 and 2590 MBq, respectively) had an age-appropriate dose reduction of ^131^I-omburtamab by 33%. One patient at dose level 1850 MBq was replaced because she experienced rapidly progressive melanoma precluding monitoring for the full DLT period. Only 5 patients were treated at 2960 MBq because there were no DLTs encountered, and because the RP2D was declared at that point.

**Table 1:** Demographics and dose escalation for the phase I study

^131^I-omburtumab dose (MBq)	Number planned in the absence of DLT	Number of patients enrolled	No. receiving at least one Rx dose	Number of NB	Number of other diagnoses**	Median actual dose received (mCi/MBq)	Number of patients treated with 2 cycles
370	3	5^c^^,d^	3	1	2	10/370	1
740	3	3	3	1	2	20.6/762.2	3
1110	3	6^1^	6^b^	5	1	30.3/1121.1	4
1480	3	3	3	1	2	40/1480	2
1850	3	6^c^^,e^	6	3	3	49.6/1835.2	3
2220	3	4^e^	4	3	1	58.1/2149.7	1
2590	6	6^a^	6	1	5	69.9/2586.3	2
2960	6	5	5	0	5	79.4/2937.8	1
Total	30	38	36	15	21		17

*DLT* dose-limiting toxicity, *NB* neuroblastoma

^a^One patient at this dose level received age-based dose reduction per protocol

^b^One patient at this dose level with DLT

^c^One patient replaced because treatment dose was not administered due to rapid disease progression after the first imaging dose

^d^One patient replaced because treatment dose was not administered due to non-compliance after the first imaging dose

^e^Additional patients treated on compassionate basis after IRB approval

^**^Other diagnoses (*n* = 21) included medulloblastoma (*n* = 6), ependymoma, (*n* = 3), melanoma and rhabdomyosarcoma (*n* = 3 each), choroid plexus carcinoma (*n* = 2), and atypical rhabdoid teratoma, chordoma, pineoblastoma and retinoblastoma (*n* = 1 each)

### Adverse events

Overall cRIT was well tolerated with the commonest adverse event being myelosuppression most commonly seen in patients who had been treated with prior craniospinal radiation therapy. Grade 4 thrombocytopenia (*n* = 7) was first observed at a median of 26 (range 23–42) days post-^131^I-omburtamab, whereas the corresponding time for grade 4 neutropenia (*n* = 4) was 39 (range 28–50) days. Adverse events during the observation period related to cRIT are summarized in Table 2. Only one DLT was encountered: transient grade 3 AST/ALT elevation in 1/6 patients at dose level 3; this patient was not known to have preexisting hepatic insufficiency; nevertheless, hepatic transaminitis was self-limited and normalized without intervention. Non-myelosuppressive MTD was not reached. Prolonged thrombocytopenia was encountered in a majority of patients receiving > 1850 MBq. Although this did not constitute a DLT, 4/6 (67%) patients receiving ^131^I-omburtamab post-CSI had grade 4 thrombocytopenia and had not attained platelet transfusion independence before the second dose would have been due. In contrast, none of the 3 patients who received 1850 MBq ^131^I-omburtamab after CSI developed prolonged thrombocytopenia. Since we envisaged combining cRIT with CSI for future therapy, especially for patients with CNS NB, 1850 MBq was declared as the RP2D.

**Table 2:** Toxicities during DLT observation period

Dose level (MBq)	N	Fever	Vomiting			Anxiety	Pruritus	Hypothermia	ALT			Bilirubin	AST			Platelets				ANC				Lymphopenia
Grade		1		1	2	1	1	2	1	2	3	1	1	2	3	1	2	3	4	1	2	3	4	3
370	3								1							1								1
740	3			1									1					1						1
1110	6	1		2				1	2	1	1		2	1	1	1			1		1			2
1480	3			1									1			1		1	0		1			
1850	6	1		1	1				1				1					1	1			2	1	
2220	4					1	1		3			1	3					1	2			1	1	1
2590	6			1					1					1		1	1		1		1		1	1
2960	5				1					1									1				1	1
Total	36	2		6	2	1	1	1	8	2	1	1	8	2	1	4	1	4	6	0	3	3	4	7

*DLT* dose-limiting toxicity, *ALT* alanine transaminase, *AST* aspartate aminotransferase, *ANC* absolute neutrophil count,

N: number receiving at least one therapeutic dose of radioiodinated omburtamab

Although the toxicity scoring period was defined as the first 5 weeks after the first cRIT dose, patients who received a second cycle of cRIT were also observed for an additional 35 days (or until PD, or until they were removed to receive other therapies). Again, treatment was well tolerated; salient toxicities are summarized in Table 3. One patient at dose level 2590 MBq developed grade 3 chemical meningitis manifesting as headache, fever, vomiting and sterile CSF pleocytosis after the second imaging dose. Therapy with supportive care and systemic steroids resulted in the complete resolution of meningitis within 3 days. The therapeutic dose was not administered to this patient. Two adult patients at dose level 8 (2960 MBq) were admitted for grade 3 nausea, dehydration and transient grade 2 hypertension, respectively. Three patients underwent elective stem cell reinfusion: two to facilitate the continuation of chemotherapy for systemic disease and one because of prolonged thrombocytopenia. Reasons for not receiving 2 cycles included progressive disease (*n* = 12), prolonged myelosuppression (*n* = 4), non-myelosuppressive toxicity (*n* = 2), and patient preference (*n* = 1). HAMA developed in 8/19 (42%) patients who were seronegative at entry.

**Table 3:** Salient toxicities in patients receiving 2 cycles

Dose level (MBq)	N	Fever	Vomiting			Headache	ALT			Bilirubin	AST			Platelets				ANC			
Grade		2		1	2	2	1	2	3	1	1	2	3	1	2	3	4	1	2	3	4
370	1																				
740	3									1							1				
1110	4	1			1												2				2
1480	2				1	1											1				1
1850	3				1	1					1					1	1			1	1
2220	1					1		1													
2590	2							1		1											
2960	1				1			1									2				2
Total	17	1		0	4	3	0	3	0	2	1	0	0	0	0	1	7	0	0	1	6

*AST* aspartate aminotransferase, *ANC* absolute neutrophil count,

### Radioimaging and dosimetry

In general, prominent activity was seen in the ventricles and the thecal space 2–4 h post-injection, with clearance from the ventricles and thecal space 24–48 h post-injection (Fig. 1). All patients had prominent activity in the ventricles showing the adequate distribution of tracer throughout the thecal sac, clearing by 24–48 h after injection. Low activity was observed on scans in the liver, kidney and/or bladder in some patients at 24–48 h (Fig. 1). Median ± SD radiation dose to the CSF and blood by sampling (*n* = 33) was 1.01 ± 1.08 and 0.04 ± 0.08 mGy/MBq, respectively, showing a consistently high therapeutic advantage for CSF versus blood. Median and mean CSF/blood radiation dose ratios were 24.2 and 77.1. By nuclear imaging (*n* = 23 patients), ventricular and CSF radiation doses were 1.16 ± 0.54 and 1.04 ± 0.95 mGy/MBq, respectively. There was a moderate correlation between CSF radiation dose estimated by tissue sampling versus by nuclear imaging (*r* = 0.54). Interpatient variability estimated by CSF tissue sampling was higher for ^123^I-omburtamab versus ^131^I-omburtamab (SEM of 0.03 vs 1 mGy/MBq), while the interpatient variability based on nuclear imaging was comparable for ^124^I-omburtamab PET versus ^131^I-omburtamab SPECT (SEM 0.03 vs 0.06). There was wide interpatient variability for CSF/blood radiation dose ratios, but no obvious relationship to the administered dose. However, there appeared to be a trend for higher calculated CSF absorbed dose for administered activity ≥ 2220 MBq (median 1.37 mGy/MBq) versus those < 2220 MBq (median 0.89 mGy/MBq). Mean cumulative absorbed CSF and blood dose for all administrations (*n* = 34 patients) was 24.8 Gy (range 314–7152 cGy) and 100 cGy (15–236.9), respectively, where the differences were accounted for by the number of doses given (Table 4). Absorbed dose for major organs (*n* = 16) was well below the maximum tolerated radiation dose levels (Additional file 3: Table S2).

**Table 4:** Absorbed radiation doses and CSF/blood ratios

	Ratios	T test
*Dose 1 Sampling *(*n* = 33)		
CSF (mGy/mCi)	1.01 (0.46–4.6)	
Blood (mGy/mCi)	0.04 (0.01–0.24)	
CSF/blood ratio	24 (3–767)	
*Dose 1 Scans* (*n* = 23)		
Brain(mGy/mCi)	1.16 (0.2–3.75)	
CSF (mGy/mCi)	1.04 (0.25–2.84)	
*Dose 2 Sampling*		
< 2220 MBq (*n* = 20) CSF	0.89 (0.34–2.18)	0.09*
> 2220 MBq (*n* = 13)	1.37 (0.76–3.98)	
< 2220 MBq (*n* = 20) CSF/blood ratio	38.2 (4.8–59.3)	0.02*
> 2220 MBq (*n* = 13)	50.7 (14.9–314.2)	
	Absorbed radiation dose (rads)	
*Cumulative dose for all administrations*		
CSF	1672 (336.2–8966)	
Blood	82 (15–236.9)	

*CSF* cerebrospinal fluid

*t test comparing patients with absorbed dose of < 2220 rads vs > 2220 rads

Patients with prior exposure to murine mAbs, as in the case of several patients treated with anti-GD2 antibodies for systemic neuroblastoma, often had detectable HAMA levels at the time of omburtamab treatment. The presence of HAMA and exposure to prior murine mAbs prior to study entry had a significant impact on omburtamab kinetics. Median CSF/blood ratio calculated after the first (tracer) and second (therapeutic) dose of radioiodinated omburtamab was significantly higher (*P* < 0.05) both for patients with preexisting HAMA vs those with negative HAMA and for those who had previously received murine mAbs (Table 5).

**Table 5:** Impact of prior murine antibody exposure and antidrug development on omburtamab pharmacokinetics

	No. of patients tested	CSF/blood ratio				t test
		Med	STDEV	Mean	SEM	
*Dose 1*						
HAHA neg	16	22.3	32.9	36.3	2.1	0.04*
HAHA pos	11	54.2	239.3	162.6	21.8	
No prior Ab	17	19.8	22.9	25.5	1.4	0.02**
Prior Ab	14	55.9	213.1	144.6	15.2	
*Dose 2*						
HAHA neg	19	20.4	20.3	25.0	1.1	0.04*
HAHA pos	10	28.1	99.9	76.9	2.8	
No prior Ab	15	14.9	15.0	19.9	1.0	0.05**
Prior Ab	14	33.7	85.5	65.4	2.4	

*Med* median, *STDEV* standard deviation from the mean, *SEM* standard error of the mean, *HAHA* human antihuman antigen, *Ab* antibody

^*^t test comparing HAHA-negative vs HAHA-positive patients

^**^t test comparing patients with prior mAb exposure vs those without

### Response and Survival

Response assessment was not a primary endpoint of this phase I study, indeed only 17 patients had evaluable or measurable disease prior to cRIT, only 2 of whom had not received prior radiotherapy. Of the latter, 1 patient had mild radiological improvement, and the other had stable findings on follow-up MRI (Additional file 3: Table S3). Three patients (none with NB) had malignant cells on CSF cytology immediately prior to therapy. These persisted after cRIT. In patients with NB (*n* = 15) receiving cRIT as part of a multimodality salvage therapy, a significant improvement in overall survival and progression-free survival was noted when compared to historical data (Additional file 3: Table S4). Seven 7 (47%) survive without progressive disease 13–17 years from cRIT. Of the remaining 8, only 2 experienced a further CNS relapse and the remaining 6 were dying of systemic relapse or chemotherapy-related toxicity. For patients with NB, median PFS, CNS PFS and OS were 7.5, 14.1 and 11.1 years, respectively (Additional file 2: Fig. S2). Because long-term survivors of CNS neuroblastoma relapse were observed at each dose level, it did not appear that outcome was related to the dose level or the number of doses administered (*P* > 0.1 for each); however, patients with isolated CNS relapse had a significantly better outcome than those with CNS and systemic relapse (*P* < 0.05) (Additional file 3: Table S4). This implies that even 370 MBq ^131^I-omburtamab may be sufficient in some patients to eradicate micrometastases. For patients with diagnoses other than NB, RIT was not associated with as favorable an impact with median PFS, CNS PFS and OS of 0.2, 1 and 1 year, respectively. However, 6 patients with relapsed non-NB diagnoses survived > 5 years after RIT including 2 with ependymoma and 1 each with medulloblastoma, chordoma, choroid plexus carcinoma and ETMR. The latter 3 continue to survive disease-free 7.4 + , 9.3 + and 11.7 years after RIT including 2 who had not received radiotherapy to the neuraxis.

## Discussion

The B7 family of checkpoint regulators has energized cancer immunotherapy; however, the immunoregulatory role of B7H3 specifically remains elusive, and it has not been as successfully targeted by immunotherapies as other related molecules. In previous preclinical and clinical studies, we have shown that B7H3 is a tumor antigen that can be targeted for radioimmunotherapy using radioiodinated omburtamab, a murine anti-B7H3 antibody [7, 16, 17, 25]. We have also shown that B7H3 is abundantly and homogenously expressed in a wide range of embryonal tumors with limited expression on normal tissues, making it an attractive tumor target [19]. We now show that anti-B7H3 monoclonal antibody omburtamab can be safely used for cRIT even in patients who had previously received high doses of chemotherapy and radiotherapy to extensive fields, as is the case for patients with relapsed CNS neuroblastoma or medulloblastoma post craniospinal irradiation [13]. Related adverse events were modest, and therapy could easily be done outpatient. We did encounter the expected myelosuppression with the use of a radioiodinated antibody, but significant thrombocytopenia was noted predominantly in those patients previously treated with CSI which significantly affects bone marrow reserve. The recommended phase II dose of 1850 MBq was chosen in view of the myelosuppression noted at higher doses in patients previously treated with chemotherapy and CSI, since we anticipated that they would constitute most of the subjects in phase II omburtamab trials.

The CSF space is uniquely suited to compartmental radioimmunotherapy for leptomeningeal malignancies. It is not amenable to intravenous antibody therapy because of the blood–brain barrier; conversely, the transfer of antibodies between the CSF and the meninges is relatively free [26]. The compartmental approach successfully avoids early clearance of the drug in the hepatic circulation, anticipated with intravenous drug administration of a murine monoclonal antibody. Moreover, since CSF does not recirculate, has few circulating cells, and has low protein levels, it is amenable to the modeling of antibody circulation and distribution. A two-compartment pharmacokinetic modeling method fitted to the radioactivity measurements in the CSF derived from serial sampling from 61 patients receiving ^131^I-omburtamab on this phase I and extension phase II studies supported the fractionation of ^131^I-omburtamab cRIT into two doses to optimize therapeutic index [27]. In this phase I study, we were able to administer two therapeutic doses of cRIT safely in the majority of patients able to receive such therapy without PD after the first dose. The CSF-to-blood ratio was high suggesting a high therapeutic index. Interestingly, this ratio was further increased in patients who had a preexisting HAMA titer, having previously received murine antibodies for NB therapy [28]. This increase in therapeutic index indicates that HAMA accelerates the clearance of antibody from the blood but not from the CSF. The emergence of HAMA can negatively impact the PK of intravenously injected antibodies by accelerating systemic clearance and diminishing targeting to tumor. However, the presence of HAMA can be an advantage when body compartments such as the CSF and peritoneum are targeted. Indeed, HAMA seropositivity could enhance off-target systemic clearance without impacting ventricular and thecal sac exposure as has been observed in preclinical studies in non-human primates [9]. This observation confirmed what had been noted in preclinical studies, namely that HAMA seropositivity could enhance off-target systemic clearance without impacting ventricular and thecal sac exposure [9].

^124^I-omburtamab allows the use of precise PET imaging to accurately determine radiation exposure of normal organs to cRIT. We have used this approach to demonstrate minimal exposure to normal organs when ^124^I-omburtamab is injected into the CSF [22] or into the peritoneum [16]. Radioimmuno-PET imaging allowed the determination of exposure to organs, all of which received doses far below tolerability in cRIT. Although the average dose to red marrow was fairly consistent in all patients, the CSF absorbed dose delivered by cRIT was more variable, likely reflecting multiple factors such as CSF flow differences secondary to surgical insults, radiation, and presence or absence of bulk lesions. Since most patients were treated with cRIT as consolidation for micrometastases, dosimetry of small tumor volume was difficult to assess and the therapeutic effect of radioiodinated mAb was even harder to isolate. This variability in CSF mGy/MBq ratios across many dose levels also made an ideal CSF target dose difficult to assess.

While response and survival were not primary endpoints for this study, we observed an impressive long-term survival for patients with CNS relapse of NB. cRIT was an important component of multimodal salvage therapy for these patients [7], and 44% (7/16) of patients are long-term survivors 13–17 years after cRIT and only 13% (2/16) experienced a further CNS relapse after receiving cRIT. This compares extremely favorably to published data on CNS relapse of NB who have 100% mortality with a median survival of less than 6 months [29, 30]. Although all patients who are long-term survivors had also received CNS radiotherapy, these encouraging outcomes have not previously been reported suggesting at least an additive effect for cRIT. More importantly, the incidence of radionecrosis was low among long-term survivors despite their significant prior therapies [31]. Based on these encouraging data, we have incorporated cRIT ^131^I-omburtamab into salvage therapy for relapsed NB [7] and have recently completed the phase II study in which a large number of patients with CNS relapse of NB have been treated [32]. In addition, we note extended survival for several histologic diagnoses of several tumors treated with focal radiation therapy and ^131^I-omburtamab including ETMR [33] and Li-Fraumeni-associated recurrent choroid plexus carcinoma.

## Conclusion

We demonstrated that cRIT targeting B7H3 was safe and could be incorporated into multimodality therapies for patients with leptomeningeal malignancies. The therapeutic index between CSF and blood was highly favorable and myelotoxicity tolerable. There appeared to be a benefit on the therapeutic index if there were neutralizing antibodies in the blood to accelerate systemic clearance. The long-term survival in a subset of patients was totally unexpected.

## Supplementary Information

**Additional file 1**.** Figure S1**. Consort diagram.

**Additional file 2**.** Figure S2**. Overall survival in patients with neuroblastoma and other diagnoses.

**Additional file 3**. Tables S1–S4.

## Data Availability

The protocol summary, a statistical summary, and informed consent form will be made available on clinicaltrials.gov when required as a condition of Federal awards, other agreements supporting the research and/or as otherwise required. Requests for deidentified individual participant data can be made beginning 12 months after publication and for up to 36 months post-publication. Deidentified individual participant data reported in the manuscript will be shared under the terms of a Data Use Agreement and may only be used for approved proposals. Requests may be made to: crdatashare@mskcc.org. Requests should contain adequate information on the type of analysis to be performed and a full listing of parties that will have access through this request.

## Abbreviations

Ab: Antibody
ALT: Alanine transaminase
AST: Aspartate aminotransferase
ANC: Absolute neutrophil count
BM: Bone marrow
CNS: Central nervous system
cRIT: Compartmental radioimmunotherapy
CSF: Cerebrospinal fluid
CSI: Craniospinal irradiation
CTCAE: Common Terminology Criteria for Adverse Events
CTCAP: Computed tomography of the chest, abdomen, and pelvis
DLT: Dose-limiting toxicity
e +: Emitting positron
HAHA: Human antihuman antibody
HAMA: Human anti-mouse antibody
mAb: Monoclonal antibody
MBq: Megabecquerels
Gy: Gray
MIBG: Meta-iodobenzylguanidine
MTD: Maximum tolerated dose
NB: Neuroblastoma
OS: Overall survival
PET: Positron emission tomography
PFS: Progression-free survival
PK: Pharmacokinetics
PLTS: Platelets
SPECT: Single-photon emission computed tomography
