# Supplementary figures and images for: Phase 1 study of intraventricular 131I-omburtamab targeting B7H3 (CD276)-expressing CNS malignancies
Source: J Hematol Oncol. 2022 Nov 12;15:165. doi: 10.1186/s13045-022-01383-4 (PMC9655863; doi:10.1186/s13045-022-01383-4)

Supplemental figure S1. Consort diagram.

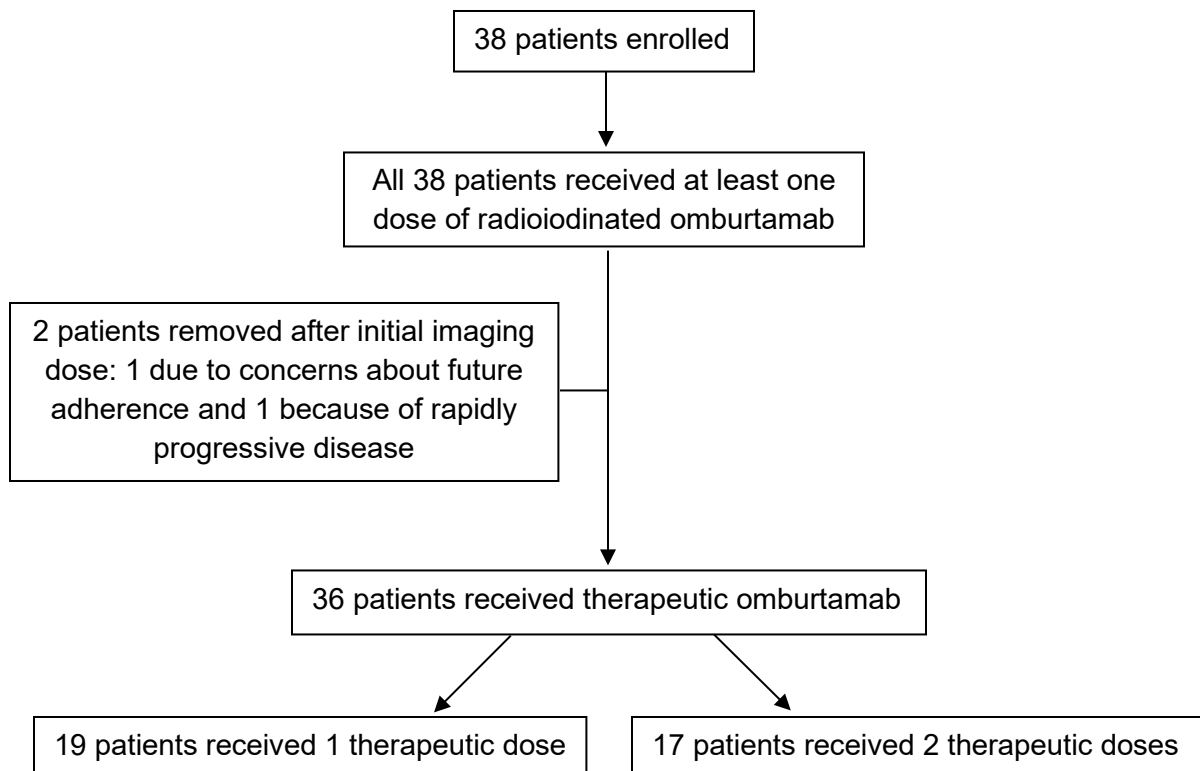

Supplement: Supplementary file 1 — Additional file 1. Figure S1. Consort diagram. [file 13045_2022_1383_MOESM1_ESM.pdf]

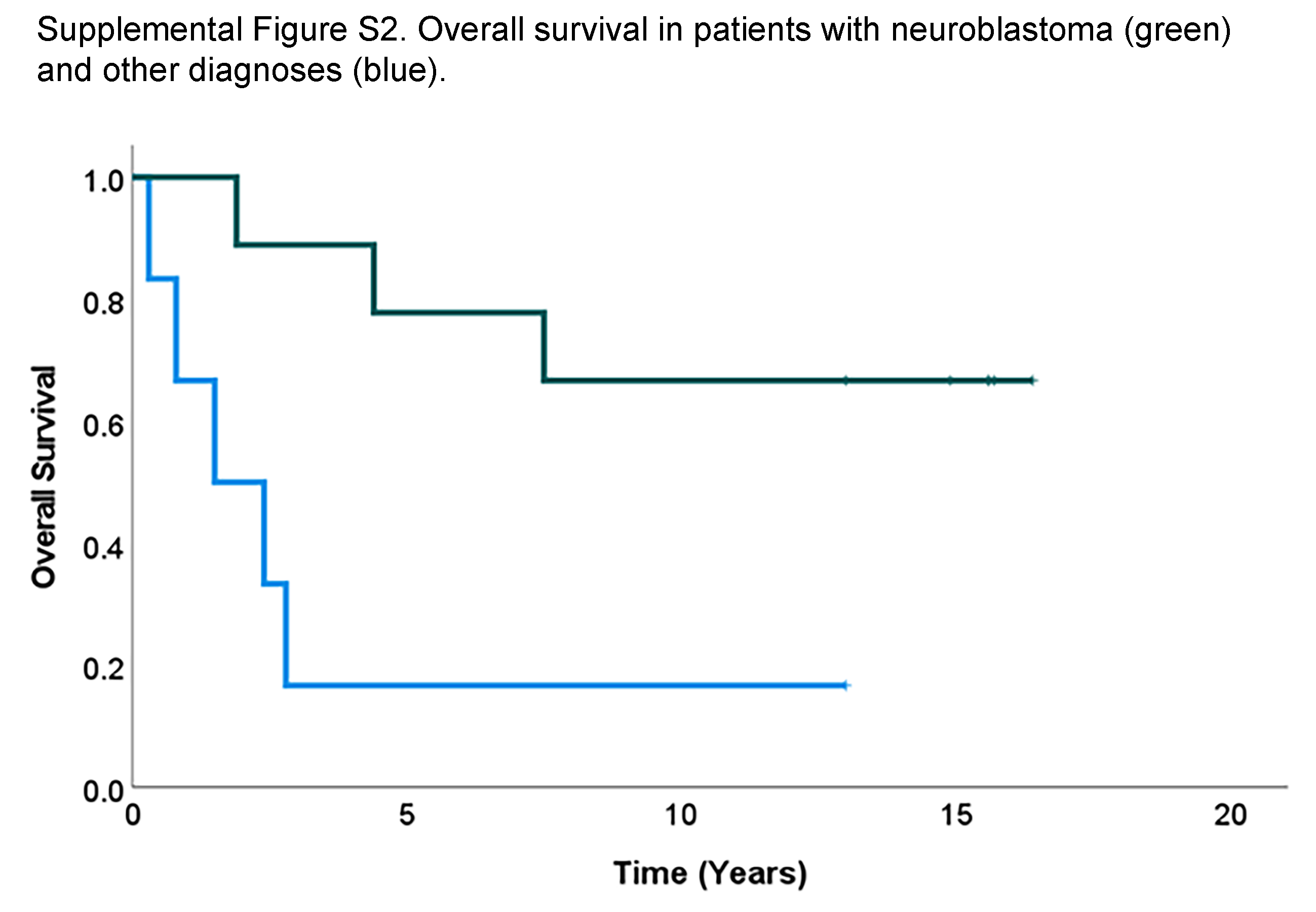

Supplement: Supplementary file 2 — Additional file 2. Figure S2. Overall survival in patients with neuroblastoma and other diagnoses. [file 13045_2022_1383_MOESM2_ESM.tif]
