# Supplementary material for: Phase 1 study of intraventricular 131I-omburtamab targeting B7H3 (CD276)-expressing CNS malignancies
Source: J Hematol Oncol. 2022 Nov 12;15:165. doi: 10.1186/s13045-022-01383-4 (PMC9655863; doi:10.1186/s13045-022-01383-4)
Supplement: Supplementary file 3 — Additional file 3. Tables S1–S4. [file 13045_2022_1383_MOESM3_ESM.docx]

Supplemental Table S1. Treatment schema

| Day | Treatment/Intervention |
| --- | --- |
| Pre-8H9 | ^111^In-DTPA scan for Ommaya patency and CSF flow |
| -14- +14 | Oral liothyronine and/or potassium iodide for thyroid protection |
| -1 | IV Dexamethasone |
| 0 | Dosimetric dose (74MBq) of ^124^I or ^131^I -omburtamab cRIT. Blood draw for 8H9 pharmacokinetics. |
| 1-2 | Blood draw for ^124^I-8H9 pharmacokinetics. PET scan for ^124^I-8H9 dosimetry. |
| 7 | Therapeutic dose of ^131^I -omburtamab cRIT |
| 3-7 | Blood draw for ^131^I-8H9 pharmacokinetics. Gamma camera scan for ^131^I-8H9 distribution. |
| 0-35 | Observation for DLT |
| Week 5 | Follow up scans |

DTPA, Diethylenetriamine pentaacetate; CSF, cerebrospinal fluid; cRIT, compartmental radioimmunotherapy; PET, positron emission tomography; DLT, dose limiting toxicity.

Supplemental Table S2. Absorbed doses for major organs.

| **OLINDA Mean Absorbed Doses (mGy/MBq)** | | | |  | **Mean** | **SD** | **Median** | **Min** | **Max** |
| --- | --- | --- | --- | --- | --- | --- | --- | --- | --- |
| **CSF** |  |  |  |  | 1.94 | 0.61 | 1.95 | 0.95 | 2.84 |
| **Salivary Gland** |  |  |  |  | 0.24 | 0.16 | 0.25 | 0.07 | 0.49 |
| **Adrenals** |  |  |  |  | 0.26 | 0.19 | 0.22 | 0.06 | 0.67 |
| **Brain** |  |  |  |  | 1.20 | 0.64 | 1.07 | 0.20 | 2.62 |
| **Small Intestine** | |  |  |  | 0.24 | 0.19 | 0.20 | 0.06 | 0.69 |
| **Stomach Wall** | |  |  |  | 0.44 | 0.24 | 0.37 | 0.19 | 0.84 |
| **Heart Wall** | |  |  |  | 0.24 | 0.18 | 0.20 | 0.06 | 0.66 |
| **Kidneys** |  |  |  |  | 0.24 | 0.18 | 0.20 | 0.06 | 0.65 |
| **Liver** |  |  |  |  | 1.05 | 0.99 | 0.92 | 0.12 | 3.52 |
| **Lungs** |  |  |  |  | 0.22 | 0.17 | 0.19 | 0.05 | 0.63 |
| **Red Marrow** | |  |  |  | 0.22 | 0.22 | 0.17 | 0.06 | 0.81 |
| **Spleen** |  |  |  |  | 0.33 | 0.25 | 0.24 | 0.04 | 0.76 |
| **Thyroid** |  |  |  |  | 0.28 | 0.27 | 0.17 | 0.07 | 0.97 |
| **Urinary Bladder Wall** | | |  |  | 0.22 | 0.17 | 0.18 | 0.06 | 0.64 |
| **Total Body** | |  |  |  | 0.28 | 0.21 | 0.23 | 0.08 | 0.78 |

OLINDA, organ level Internal Dose Assessment; mGy, milligray; MBq, megabecquerel; SD, standard deviation; Min, minimum; Max, Maximum; CSF, cerebrospinal fluid

Supplemental Table S3. Responses

|  | Prior WB/CSI | End of treatment response | No prior WB/CSI | End of treatment response |
| --- | --- | --- | --- | --- |
| NB (n=8) | 5 | 2: PD; 4: SD | 1 | SD |
| Non-NB (n=11) | 10 | 6 PD; 2 SD; 2 SD | 1 | SD |

NB, neuroblastoma; WB/CSI, whole body/craniospinal irradiation; PD, progressive disease; SD, stable disease

Supplemental Table S4. Survival

| All |  | p | (years) | p | PFS (years) | p | CNSPFS (years) |
| --- | --- | --- | --- | --- | --- | --- | --- |
| NB | 16 | 0.01 | 7.5 | 0.01 | 11.1 | 0.01 | 14.1 |
| Non NB | 21 |  | 1 |  | 0.2 |  | 1 |
|  |  |  |  |  |  |  |  |
| **NB only** |  |  |  |  |  |  |  |
| 1 Dose | 7 | 0.8 | 7.5 | 0.45 | 14.7 | 0.73 | 14.6 |
| 2 Doses | 8 |  | 4.4 |  | 17.2 |  | 17.9 |
|  |  |  |  |  |  |  |  |
| Isolated | 9 | 0.01 | 16.2 | 0.02 | 17.9 | 0.03 |  |
| Disseminated | 6 |  | 6.9 |  | 9.4 |  |  |
|  |  |  |  |  |  |  |  |
| Dose <60 | 8 | 0.16 | 15.9 | 0.06 | 18 | 0.83 | 17.6 |
| Dose >/=60 | 7 |  | 9.4 |  | 10.4 |  | 18.2 |

NB, neuroblastoma; PFS, progression free survival; CNSPFS, central nervous system progression free survivial
